# A novel protein kinase inhibitor IMB-YH-8 with anti-tuberculosis activity

**DOI:** 10.1038/s41598-017-04108-7

**Published:** 2017-07-11

**Authors:** Jian Xu, Ju-xian Wang, Jin-ming Zhou, Chang-liang Xu, Bin Huang, Yun Xing, Bin Wang, Rui Luo, Yu-cheng Wang, Xue-fu You, Yu Lu, Li-yan Yu

**Affiliations:** 1 Institute of Medicinal Biotechnology, Chinese Academy of Medical Sciences and Peking Union Medical College, 1# Tian Tan Xi Li, Chongwen district, Beijing, 100050 China; 2 Beijing Key Laboratory of Drug Resistance Tuberculosis Research, Beijing Tuberculosis and Thoracic Tumor Research Institute, Beijing Chest Hospital, Capital Medical University, Beijing, 101149 China

## Abstract

Protein kinase B (PknB) is one of the *Mycobacterium tuberculosis* serine/threonine protein kinases and has an essential role in sustaining mycobacterial growth. Here, we identified and characterized a novel small molecule compound named IMB-YH-8 that inhibited PknB and served as anti-mycobacteria lead compound. IMB-YH-8 inhibited PknB auto-phosphorylation and the phosphorylation of GarA by PknB in a dose-dependent manner. The compound did not inhibit human Akt1 or other serine/threonine kinases in *M*. *tuberculosis* except for the highly homologous PknA. IMB-YH-8 bound to PknB with a moderate affinity. Molecular docking revealed that IMB-YH-8 interacts with the catalytic domain of PknB. Observations of electron microscopy showed that IMB-YH-8 changed the morphology of H37Rv and disrupted the cell wall. The differential transcriptional response of *M*. *tuberculosis* to IMB-YH-8 revealed changes in SigH regulatory pathways modulated by PknB. Notably IMB-YH-8 not only potently inhibited drug-sensitive and multidrug-resistant clinical isolates but also exhibited a dose dependent inhibition of intracellular *M*. *tuberculosis*. Taken together, these *in vitro* data demonstrate that IMB-YH-8 is a novel inhibitor of PknB, which potently prevents growth of *M*. *tuberculosis*. It is as yet unclear whether inhibition of PknA contributes to the anti-tubercular action of IMB-YH-8.

## Introduction

Tuberculosis (TB) remains one of the most deadly infectious diseases in the world. There were an estimated 10.4 million incident TB cases and 1.4 million died of this disease in 2015 alone according to the report by World Health Organization^1^. Patients with multidrug-resistant tuberculosis (MDR-TB) or extensively drug-resistant tuberculosis (XDR-TB) need combinations of second-line and third-line anti-tuberculosis drugs, which are much more expensive, more toxic, longer in duration, and less effective compared to the first-line regimen for treating drug-sensitive tuberculosis^2, 3^. Thus, there is an urgent need to discover new drugs with novel mechanisms of action against *Mycobacterium tuberculosis* ^3, 4^.

Protein phosphorylation plays a key role in signaling transduction not only in eukaryotes but also in *M*. *tuberculosis* ^5^, which encodes 11 serine/threonine protein kinases (STPKs) including PknA, PknB, PknC, PknD, PknE, PknF, PknG, PknH, PknJ, PknK, and PknL that regulate cell division, cell development, cell metabolism and dormancy^6–8^. PknB is a trans-membrane protein, conserved among Gram-positive bacteria and essential for mycobacterial viability^9^. PknB is predominantly expressed during exponential growth. Partial depletion or overexpression of *pknB* causes morphological changes associated with defects in the cell wall synthesis and in cell division^6, 9^. Recent findings showed that PknB is essential for both *in vitro* growth and survival of *M*. *tuberculosis* in the host^10^. PknB mediates an oxygen-dependent replication switch. All these studies suggest that PknB could be a drug target for developing therapies to inhibit both the active and latent forms of tuberculosis^11^. Furthermore, the PknB kinase domain exhibits less than 30% similarity to eukaryotic kinases^5, 12^, which suggests that PknB inhibitors may be specific for bacteria and not affect the host kinases.

In our previous studies, a high throughput screening assay was established to discover inhibitors of *M*. *tuberculosis* PknB. Eight out of 18 000 compounds showed an inhibitory effect on PknB, among which 3 compounds including IMB-YH-8 inhibited *Mycobacterium marinum* and *Mycobacterium smegmatis* ^13^. In this study, we further demonstrate that IMB-YH-8 suppresses both drug-sensitive and drug-resistant *M*. *tuberculosis* strains. IMB-YH-8 inhibits auto-phosphorylation and substrate phosphorylation of PknB with good specificity. The interaction between IMB-YH-8 and PknB was determined in the binding affinity experiments and docking study. Gene expression profiles show that IMB-YH-8 modulates PknB and SigH regulatory pathways.

## Results

### IMB-YH-8 inhibits auto-phosphorylation and substrate phosphorylation activities of PknB

**Figure 1:**
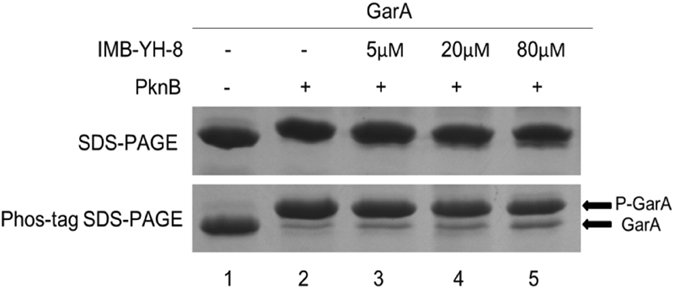
*In vitro* activity of IMB-YH-8 in substrate phosphorylation of GarA by PknB. Upper panel: Phosphorylated and non-phosphorylated GarA proteins were analyzed on 12% SDS-PAGE alone; Lower panel: Phosphorylated and non-phosphorylated GarA proteins were separated on 12% SDS-PAGE containing 20 μM Phos-tag acrylamide and 100 µM MnCl_2_. (full-length gels are presented in Supplementary Figure 1)

Results of our high throughput screening assay revealed IMB-YH-8 (C_12_H_12_O_4_, MW: 220.07) as an inhibitor of *M*. *tuberculosis* PknB^13^. To confirm IMB-YH-8 inhibition of PknB, we measured the effect of IMB-YH-8 on PknB auto-phosphorylation and PknB-mediated phosphorylation of GarA. PknB is activated by auto-phosphorylation of Ser and Thr residues to control phospho-signaling pathways^14–16^. In the non-radioactive assay, IMB-YH-8 inhibited auto-phosphorylation of PknB with an IC_50_ of 20.2 μM (Table 1). In order to assess the effect of IMB-YH-8 on substrate phosphorylation of PknB, a Forkhead-associated (FHA) domain-containing protein (GarA) was utilized as the substrate of PknB in the phosphorylation assay. It is known that PknB efficiently phosphorylates GarA at a single N-terminal threonine residue Thr22^17^. Phosphorylated and non-phosphorylated GarA proteins were separated using SDS-PAGE containing Phos-tag acrylamide_._ Phos-tag acrylamide provides a phosphate affinity SDS-PAGE for mobility shift detection of phosphorylated proteins. In the presence of MnCl_2_, phosphorylated proteins migrate slower than the non-phosphorylated form due to phosphate trapping by the Phos-tag chemical^18^. While use of SDS-PAGE alone produced the equal amount of GarA protein, samples separated on SDS-PAGE containing 20 μM Phos-tag showed two discernible bands (Fig. 1). In lane 1, the non-phosphorylated GarA forms a lower band, while in lane 2, ATP-treated GarA was phosphorylated and showed as an upper band (Fig. 1). Treatment with IMB-YH-8 increased the signal of the non-phosphorylated GarA band, indicating that IMB-YH-8 inhibited the PknB-catalyzed phosphorylation of GarA. The level of non-phosphorylated GarA increased in a dose-dependent manner (Fig. 1, lanes 3–5). Taken together, these results demonstrate that IMB-YH-8 could inhibit both auto-phosphorylation (Table 1) and substrate phosphorylation by PknB.

**Table 1:** Structure, Log*P*, enzyme inhibition activity against PknB, anti-tuberculosis activity, and selectivity of IMB-YH-8.

Compound	Structure	Log*P* ^a^	PknB IC_50_ (μM)	H37Rv MIC (μg/ml)	THP-1 cell CC_50_ (μg/ml)	SI^b^
IMB-YH-8		2.022	20.2	0.25	7.61	30.4

^a^Log*P* determined with Discovery Studio 2.5. ^b^SI, selectivity index, calculated as the CC_50_/MIC.

### IMB-YH-8 selectively inhibits PknB and PknA without affecting other STPKs

The STPKs share a well-conserved catalytic scaffold^12, 16^. In order to investigate the specificity of IMB-YH-8 in inhibiting serine/threonine kinases, we tested the effect of IMB-YH-8 on the auto-phosphorylation activity of 5 *M*. *tuberculosis* STPKs (PknA, PknB, PknG, PknF and PknH) and human kinase Akt1. It was observed that IMB-YH-8 did not affect PknG, PknH, PknF or Akt1 activity (Table 2). Not surprisingly, IMB-YH-8 inhibited PknA at an IC_50_ of 44.3 μM which is two-fold higher than the IC_50_ of 20.2 μM measured for PknB (Table 2). This is because the kinase domains of PknA and PknB are highly conserved in sequence and structure^6, 19^. These results demonstrate that IMB-YH-8 selectively inhibits PknB and its homolog PknA of *M*. *tuberculosis*, but does not inhibit other *M*. *tuberculosis* STPKs or human serine/threonine kinase Akt1.

**Table 2:** The activity of IMB-YH-8 against *M*. *tuberculosis* and human STPKs.

Compound	IC_50_ (µM)					
	PknB	PknA	PknG	PknF	PknH	Akt1
IMB-YH-8	20.2	44.3	349.2	503.4	>640	>640

### IMB-YH-8 binds to PknB

**Figure 2:**
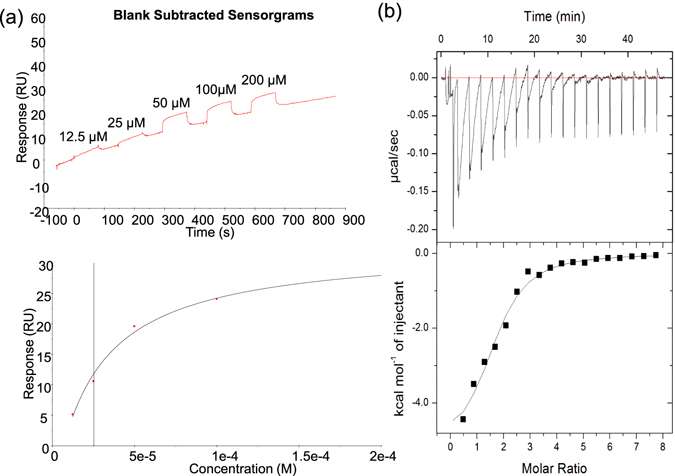
Binding of IMB-YH-8 to PknB as measured by surface plasmon resonance (**a**) and isothermal titration calorimetry (**b)**. (**a**) Upper panel: Blank subtracted sensorgram for the binding of IMB-YH-8 (range from 12.5 to 200 μM). Lower panel: The affinity for IMB-YH-8 binding (*K*_D_ = 25.3 μM) was calculated from the association equilibrium data. (**b**) Thermograms (upper panel) and binding isotherms with theoretical fits (lower panel) obtained for the binding of IMB-YH-8 to PknB.

We next performed BIAcore and ITC assays to determine whether IMB-YH-8 binds to PknB. BIAcore was designed with His capture PknB immobilized onto a CM5 sensor chip as a single cycle kinetics. As shown in Fig. 2a, IMB-YH-8 interacted with PknB in a dose dependent manner. Due to the high dissociation rates, the binding of IMB-YH-8 with PknB reached equilibrium rapidly at the association phase. The responses at equilibrium fit well with a simple 1:1 binding model. The affinity constant Kd for IMB-YH-8 binding to PknB was measured as 25.3 μM which is in close agreement with the IC_50_ value of 20.2 μM.

ITC provides the thermodynamic signature of a small molecule when it interacts with its macromolecular target in terms of enthalpy change (Δ*H*), entropy change (Δ*S*), and Gibbs free energy change (Δ*G*) along with the binding affinity constant (*Ka*) and the stoichiometry. Results of the ITC experiments showed that the binding of IMB-YH-8 to PknB was a single binding event and an exothermic process (Fig. 2b). The *K*_d_ value of IMB-YH-8 (13.5 μM) was calculated from observed *K*_a_ value, which is consistent with the data derived from SPR measurements and the IC_50_ value. Binding of IMB-YH-8 to PknB was enthalpy-driven (Δ*H* = *−*5.7 kcal/mol) together with a favorable entropic contribution (−*T*Δ*S* = −0.94 kcal/mol), which results in a Δ*G* of −6.64 kcal/mol. These data indicate that IMB-YH-8 interacts with PknB with a moderate binding affinity.

### Molecular docking reveals that IMB-YH-8 interacts with the catalytic domain of PknB

**Figure 3:**
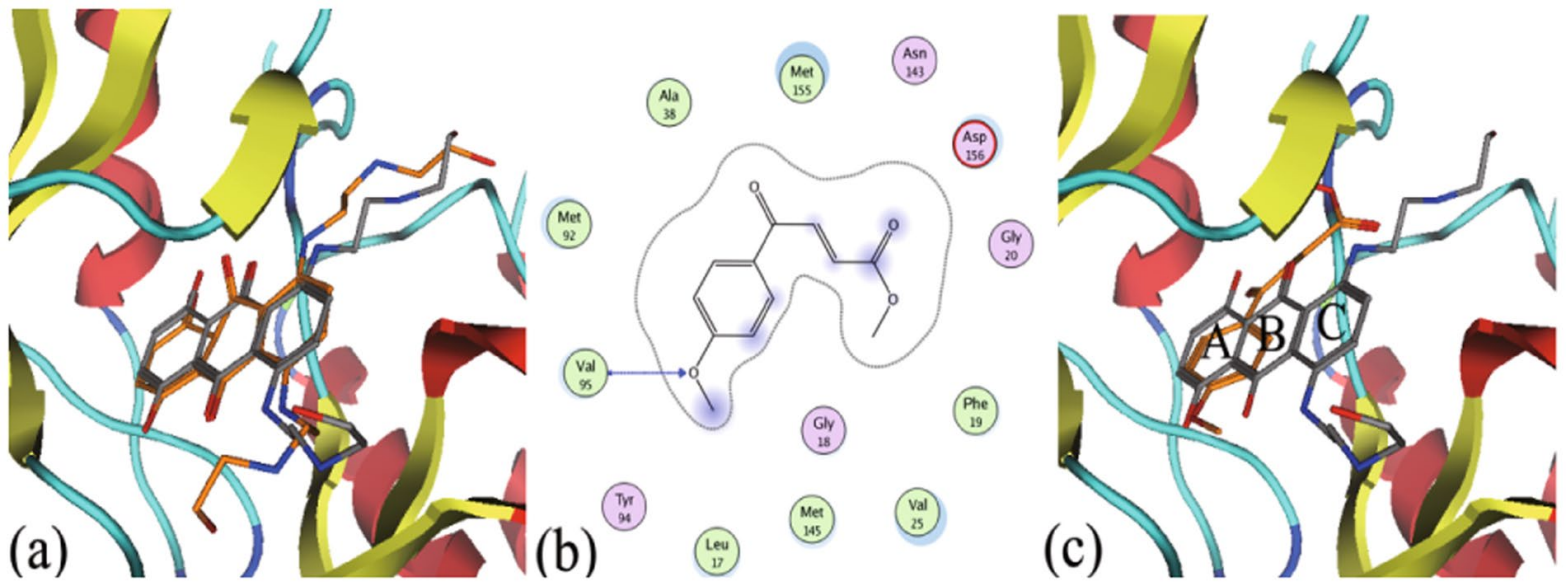
The predicted mode of IMB-YH-8 binding to PknB. (**a**) Superimposition of the predicted binding mode of mitoxantrone (in stick, carbon in orange) in the context of the crystal structure (in stick, carbon in grey). (**b**) The detailed interactions between IMB-YH-8 and PknB. (**c**) The alignment between the binding mode of IMB-YH-8 (in stick, carbon in orange) and MIX (in stick, carbon in grey). The receptor: hydrogen bond is indicated by the blue line with arrow.

We next utilized molecular docking to illustrate IMB-YH-8 binding to PknB. During the docking studies, the original ligand in the crystal structure mitoxantrone (MIX) was selected as the reference compound. The predicted binding mode by molecular docking is quite close to that in crystal ribbon structure with the RMSD value of 1.0 Å (Fig. 3a), which illustrates the reliability of the docking method. We next docked IMB-YH-8 into the binding site at the catalytic domain of PknB using the same protocols. The results (Fig. 3b and c) showed that IMB-YH-8 formed a hydrogen bond with Val95 and engaged in hydrophobic interactions with Leu17, Phe19, Val25, Ala38, Met92, Val95, Met145 and Met155. By further alignment with MIX in the binding site, we observed that the benzene ring of IMB-YH-8 matched well with the “A” ring of MIX (Fig. 3c).

### IMB-YH-8 deforms the cell wall of mycobacteria

**Figure 4:**
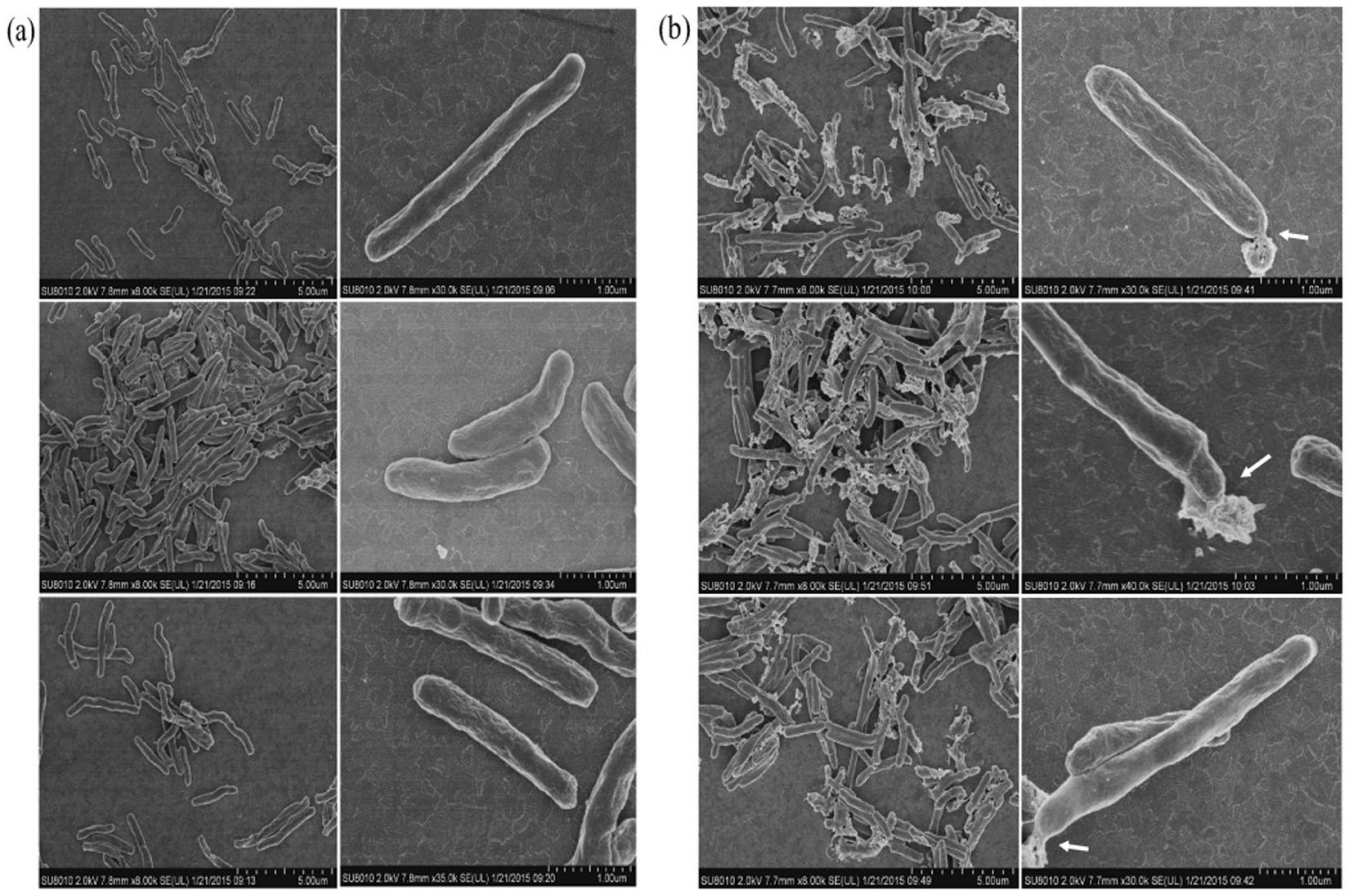
Scanning electron microscopy of untreated and 1 μg/ml IMB-YH-8 treated *M*. *tuberculosis* H37Rv for 5 days. (**a**) Untreated *M*. *tuberculosis* H37Rv (**b**) 1 μg/ml IMB-YH-8 treated *M*. *tuberculosis* H37Rv. Three independent experiments for each group are shown. Left panel, bars indicate 5 μm; right panel, bars indicate 1.0 μm. Arrows indicate changes in cell wall morphology.

PknB participates in cell shape control and cell wall synthesis. Inactivation of PknB changes cell morphology^6^. Therefore, we next measured the effect of IMB-YH-8 on cell morphology and assessed its inhibition of PknB *in vivo*. Cells were treated with IMB-YH-8 and then examined by scanning electron microscopy. While the untreated control cells displayed a rod-like shape and a smooth cell surface (Fig. 4a), treatment with IMB-YH-8 at 1 μg/ml for 5 days led to a weakening in the overall cell structure (Fig. 4b). We observed significant proportions (61.5%) of ruptured cells in 1 μg/ml IMB-YH-8 treated *M*. *tuberculosis* H37Rv (Fig. 4b).Given the role of PknB in maintaining cell morphology and keeping cell wall integrity, these data suggest that IMB-YH-8 disrupts the cell wall of mycobacteria by inhibiting PknB functions *in vivo*.

### IMB-YH-8 modulates PknB and SigH regulatory pathways

To monitor the IMB-YH-8-induced gene response profile at an early stage in the inhibitory effect of the drug, we treated logarithmically growing *M*. *tuberculosis* with IMB-YH-8 at the concentration of 2.5 μg/ml throughout the first 4 hr. Gene transcripts showing a mean change of at least twofold in the normalized intensity values were considered as differentially regulated genes following induction by IMB-YH-8. Five genes related to PknB including *trxB2* (*rv3913*), *trxC* (*rv3914*), *rv2466c*, *rshA* (*rv3221A*) and *sigH* (*rv3223c*) were found to be overexpressed during IMB-YH-8 exposure (Table 3). The whole transcriptional profiling data has been deposited to the Gene Expression Omnibus (GEO) database under the accession number GSE90858. Microarray results were validated by real-time RT-PCR analysis of the above genes from IMB-YH-8-treated and untreated H37Rv. The analysis of the up-regulated genes for the IMB-YH-8 stress condition showed a similar trend (Table 3). Among these five up-regulated genes, *rshA* showed the highest induction (>260-fold) followed by *rv2466c* (about 80-fold).

**Table 3:** Gene expression in *M*. *tuberculosis* treated by IMB-YH-8 with microarray and RT-PCR.

Systematic name	Gene	Description	Regulation	Fold induction in microarray (mean ± SD)	Real-time reverse transcription PCR	
					Normalized ΔCt (mean ± SD)	Mean fold change (2^ΔΔCt^ Value)
Rv3914	*trxC*	thioredoxin TrxC	Up	8.03 ± 0.33	2.455 ± 0.06	5.48
Rv3913	*trxB2*	thioredoxin reductase	Up	6.90 ± 0.75	2.940 ± 0.07	7.67
Rv2466c		hypothetical protein Rv2466c	Up	6.86 ± 1.93	6.325 ± 2.51	80.17
Rv3221A	*rshA*	anti-sigma factor RshA	Up	6.07 ± 0.33	8.045 ± 0.59	264.11
Rv3223c	*sigH*	ECF RNA polymerase sigma factor SigH	Up	2.92 ± 0.09	2.555 ± 0.11	5.88

### IMB-YH-8 inhibits both drug-sensitive and drug-resistant *M*. *tuberculosis*

IMB-YH-8 inhibited the growth of the *M*. *tuberculosis* strain H37Rv (MIC = 0.25 μg/ml) and has acceptable cytotoxicity against THP-1 cells with a selectivity index of 30.4 (Table 1). We further tested a panel of 14 *M*. *tuberculosis* clinical isolates with varying drug resistance profiles including those resistant to isoniazid, rifampin, streptomycin, ethambutol and ofloxacin by determining MICs using a microplate Alamar blue assay (MABA). The MICs of IMB-YH-8 for drug-resistant isolates ranged from 0.25 to 1 μg/ml (Table 4). These results indicate the potential utility of this compound to treat drug-resistant strains.

**Table 4:** *In vitro* activity of IMB-YH-8 against drug-sensitive and drug-resistant clinical isolates of *M*. *tuberculosis*.

Clinical isolates	Drug resistant	MIC (μg/ml)
31251	sensitive	0.25
4201	RIF	0.25
23443	RIF	0.5
12251	INH, RIF	0.5
20161	INH, RIF	0.5
5116	INH, RIF	0.25
12179	INH, RIF	0.25
6133	INH, RIF	0.25
7153	INH, RIF	1
3303	INH, STR	0.5
11277	INH, RIF, STR	0.5
23120	INH, RIF, STR	0.25
5120	INH, STR, EMB	0.5
5112	INH, RIF, STR, EMB	0.25
16559	STR, OFX	0.5

INH, isoniazid; RIF, rifampin; STR, streptomycin; EMB, ethambutol; ofloxacin, OFX.

### IMB-YH-8 inhibits intracellular *M*. *tuberculosis*

To test the activity of IMB-YH-8 against *M*. *tuberculosis* inside macrophages, we utilized IMB-YH-8 to treat THP-1 cells that were infected by *M*. *tuberculosis* H37Rv. After 3 days, we lysed the THP-1 cells and enumerated *M*. *tuberculosis* CFU. IMB-YH-8 showed a dose dependent inhibition of intracellular *M*. *tuberculosis* compared to the untreated control cells, i.e. reductions of 0.18, 0.62 and 1.11 (log_10_) in CFU were observed at IMB-YH-8 concentrations of 0.5, 1 and 2 µg/ml, respectively (Table 5). As shown in Table 5, with increasing concentrations (0.5, 1 and 2 μg/ml) of IMB-YH-8, the intracellular activity is significantly increased (P < 0.05), though the inhibitory effect of 0.5 μg/ml of IMB-YH-8 on CFU is not significant (p > 0.1). These results further demonstrate that IMB-YH-8 can inhibit both cell-free and intracellular mycobacteria.

**Table 5:** Activity of IMB-YH-8 against intracellular *M*. *tuberculosis* H37Rv.

Group	Log_10_CFU			
	2 μg/ml	1 μg/ml	0.5 μg/ml	Untreated
IMB-YH-8	3.92 ± 0.11**	4.41 ± 0.12*	4.85 ± 0.04	5.03 ± 0.12
INH	2.57 ± 0.14	3.14 ± 0.06	3.24 ± 0.05	

Values represent mean ± SD. **P = 0.008 compared to the 1 μg/ml treatment, *P = 0.011 compared to the 0.5 μg/ml treatment.

## Discussion

The essential role of PknB in sustaining the growth of *M*. *tuberculosis* makes it a potential drug target for developing TB inhibitors^9^. We discovered a new compound IMB-YH-8 that inhibits the activities of PknB to auto-phosphorylate itself or to phosphorylate its substrate GarA. As a result, IMB-YH-8 potently inhibits both cell-free and intracellular *M*. *tuberculosis* in a dose-dependent manner. IMB-YH-8 does not inhibit the activity of human serine/threonine kinase Akt1 and *M*. *tuberculosis* STPKs including PknG, PknF and PknH but does inhibit PknA. On the basis of sequence homology of their kinase domains, nine *M*. *tuberculosis* STPKs are classified into three distinct groups, including membrane-bound PknA/PknB/PknL, PknF/PknI/PknJ and PknD/PknE/PknH, whereas the two soluble enzymes PknG and PknK are separated from the others^5, 20^. PknA and PknB are highly conserved in *M*. *tuberculosis* and share more than 85% amino acid identity, which suggests that they act by a conserved mechanism and modify similar substrates^6^. PknB exhibits less than 30% homology with eukaryotic kinases^5, 12^. Although several Akt1 inhibitors were reported to diminish intracellular growth of MDR-TB^21^, they cause potentially serious off-target toxicities because Akt1 is involved in numerous different biological processes^22^. For example, Akt1 inhibitors increase insulin secretion and cause abnormal glucose metabolism^23^. Our study demonstrates that IMB-YH-8 is more specific for bacterial targets and may have limited off-target effects on human cells.

Docking IMB-YH-8 on the catalytic domain of PknB revealed that IMB-YH-8 formed a hydrogen bond with Val95 of PknB and that the aromatic ring of IMB-YH-8 enhanced the hydrophobic interaction with PknB. Both types of interactions are expected to increase the binding affinity of IMB-YH-8 to PknB. It is noteworthy that Val95 also forms a hydrogen bond with one hydroxyl group of PknB inhibitor mitoxantrone and also hydrogen bonds the N1 atom of adenosine in the PknB-AMPPCP complex^24, 25^. To further validate the essentiality, residues such as Val95 need to be mutated to show that their loss disrupts IMB-YH-8 binding and activity.

IMB-YH-8 causes damage in the cell wall of mycobacteria as shown by the results of electron microscopy. IMB-YH-8 treatment also leads to changes in morphology of *M*. *tuberculosis* including disruption of the cell wall. Similar observations were previously reported for *M*. *tuberculosis* that was treated with isoniazid or other compounds that target mycolic acids^26, 27^. These effects of IMB-YH-8 on cell wall and cell morphology are in agreement with the function of its target PknB in controlling cell shape and regulating cell wall synthesis that requires mycolic acids^6, 28, 29^.

PknB phosphorylates SigH and RshA *in vitro* and *in viv*o^30^. SigH plays a central role in an extensive transcriptional network that regulates oxidative and heat-stress responses in *M*. *tuberculosis* ^31^. As a stress sensor and redox switch, RshA provides a direct mechanism for sensing stress and activating transcription and regulates *sigH* negatively^30^. Under oxidative stress, SigH induces the expression of a thioredoxin-like gene *rv2466c* and the thioredoxin reductase/thioredoxin genes *trxB2/trxC* ^31^. Our transcriptional work supports that IMB-YH-8 inhibits PknB phosphorylation and modulates SigH regulatory pathways. We did attempt to determine the effect of IMB-YH-8 on an *M*. *tuberculosis pknB* deletion mutant using pMIND vector. However, this deletion proved unsuccessful. It may be attributed to stringent requiremnet of PknB expression for *M*. *tuberculosis* survival. Depletion of PknB eventually leads *M*. *tuberculosis* to cell death^10^.

In our work, IMB-YH-8 showed a moderate IC_50_ of 20.2 μM for *in vitro* kinase inhibition and inhibited *M*. *tuberculosis* with a MIC of 0.25 μg/ml (1.14 μM). However, Lougheed *et al*. found that their PknB inhibitors having much better IC_50_ values in the 20 nM range show MIC values of 16 μM to inactive range^32^. The potent anti-tuberculosis activity of IMB-YH-8 may result from its inhibition of both PknA and PknB. Both kinases are required for optimal growth of *M*. *tuberculosis* on the basis of saturation transposon mutagenesis studies^33^. Both *pknA* and *pknB* are part of an operon that encodes genes for cell shape control and cell wall synthesis in *M*. *tuberculosis* ^6^. Another possibility is that IMB-YH-8 inhibits PknB phosphorylation and then activates the enzyme cascade such as the SigH regulatory pathways *in vivo*. While Lougheed described that their PknB inhibitors may not target PknB in cells or PknB may not be bound by the inhibitors^32^. We also cannot exclude the possibility that IMB-YH-8 also targets other *M*. *tuberculosis* enzymes. Further studies are needed to confirm whether IMB-YH-8 targets PknB inside the mycobacterial cell. Nevertheless, we can conclude that IMB-YH-8 inhibits PknB and PknA with a moderate binding affinity in *in vitro* and displays anti-tuberculosis activity based on our data and discussion.

It is very likely that the methyl ester of IMB-YH-8 is hydrolyzed by mycobacterial and mammalian esterases. So we synthesized the carboxylate form of IMB-YH-8. However, it has no activity against *M*. *tuberculosis*. Recently, Zhai *et al*. found that serum C_max_ values of IMB-YH-8 following oral delivery of 200 mg/kg only reach sub-microgram/ml concentrations and fall below the therapeutic range within less than 1 hour^34^. It does not seem likely that IMB-YH-8 itself can be considered an attractive drug candidate. Despite these points, it is worth describing IMB-YH-8 as a lead compound for drug discovery.

## Methods

### Compounds

IMB-YH-8 ((E)-Methyl 4-(4-methoxyphenyl)-4-oxabut-2-enoate, C_12_H_12_O_4_, molecular weight: 220.07) was synthesized by the medicinal chemistry laboratory at the Institute of Medicinal Biotechnology with purity of >98.5%. The log*P* value was calculated using the molecular modeling software Discovery studio 2.5 (Accelrys). Isoniazid and rifampicin were obtained from Sigma–Aldrich.

### Protein expression and purification

The 279-residue kinase domain of PknB was expressed in *E*. *coli* BL21 (DE3) pLysS and purified as described^13^. The kinases PknA, PknG, PknF, PknH from *M*. *tuberculosis* H37Rv and human Akt1 were expressed with an N-terminal His6-tag in *E*.*coli* BL21 (DE3) pLysS. Recombinant GarA with His6-tag was purified by affinity chromatography and used as a kinase substrate. Briefly, the kinase expression vectors were first constructed. The transformed bacteria were grown in Luria-Bertani liquid medium containing 50 μg/ml kanamycin at 37 °C. Expression of recombinant protein was induced at OD_600 nm_ = 0.6 with 0.5 mM isopropyl b-D-1-thiogalactopyranoside (IPTG) at 20 °C for overnight. Cells were lysed using a cell disrupter (one shot, Constant Systems, UK) and the recombinant protein was purified from the soluble extract. The protein was first captured by affinity chromatography on a Ni-NTA column, eluted with an imidazole gradient (0–500 mM). Protein preparations were dialyzed against 40% glycerol in 25 mM Tris-buffered saline (pH = 7.5) and analyzed for purity using SDS/PAGE. Protein concentrations were determined by A_280 nm_, and preparations were stored at −80 °C for further use.

### *In vitro* protein kinase assay

A non-radioactive assay was performed using Promega ‘Kinase Glo’ plus Luminescent Kinase assay kit for the determination of IC_50_ values. As for PknB, the reaction was carried out in a 96-well plate in 45 μl buffer (25 mM Tris-HCl pH = 7.4, 5 mM MgCl_2_, 2 mM MnCl_2_, 3 μM PknB) containing the compound. After incubation at 4 °C for 30 min, ATP was added to the reaction buffer at the final concentration of 100 μM. The assay was conducted at 37 °C for 3 hours. The intensity of luminescence signal was determined by Multilabel Plate Reader (PE Envision) with addition of 50 μl Kinase Glo reagent. The IC_50_ values were calculated using the GraphPad Prism5 software.

### Phosphorylation assay by phos-tag analysis

*In vitro* phosphorylation of GarA (5 μM) by PknB (3 μM) was carried out in 50 μl reaction mixture (25 mM Tris-HCl (pH = 7.4), 5 mM MgCl_2_, 2 mM MnCl_2_). The reaction was initiated with the additional of 100 μM ATP and incubated at 37 °C for 3 hours. The reaction was terminated with the addition of SDS-PAGE sample buffer and heating the mixture at 100 °C for 5 min. The reaction mixtures were analyzed by 12% SDS-PAGE with or without Phos-tag SDS-PAGE to differentiate the phosphorylated and non-phosphorylated GarA. Phos-tag SDS-PAGE was performed with gel containing 20 μM Phos-tag acrylamide (Wako Pure Chemical Industries, Japan) and 100 µM MnCl_2_ according to the manufacturer’s instructions. Electrophoresis was performed at 4 °C in Tris-glycine SDS-PAGE running buffer at 30 mA for 2 h. Images were adjusted for contrast and brightness using Bio-Rad image lab software version 2.01.

### Surface plasmon resonance (SPR) assay

The measurements were performed using a BIAcore T200 (GE Healthcare) in a running buffer with an anti-His capture molecule on a CM5 sensor chip using single cycle kinetics. In brief, the carboxymethylated surface of the sensor chip CM5 was first activated with a mixture of 0.2 M N-ethyl-N-dimethylaminopropylcarbodiimide (EDC) and 50 mM N-hydroxysuccinimide (NHS). Subsequently, anti-His antibody (50 μg/ml) was injected into the flow cell 1 (FC1) and FC2 for immobilization with His Capture kit (GE Healthcare). Surfaces were blocked with a 7-min injection of 1 M ethanolamine at pH 8.5. Then purified His-tagged PknB (80 μg/ml) was injected into FC2 for immobilization on the sensor surface. No protein was injected into the FC1. His-captured PknB was stabilized by surface activation with 0.2 M EDC and 50 mM NHS for 2.5 min and blocking with 1.0 M ethanolamine (pH 8.5) for 2.5 min (cross-linking). The running buffer used for kinetic experiments contained 25 mM Tris (pH7.4), 150 mM NaCl, 5 mM MgCl_2_, 1 mM DDT, 1 mM MnCl_2_, and 5% DMSO. All experiments were performed at 25 °C at a flow rate of 30 μl/min. To analyze the binding of the compound with PknB, IMB-YH-8 at different concentrations (12.5, 25, 50, 100, 200 μM in running buffer) were injected to FC1 and FC2. Difference resonance spectra (FC2–FC1) were recorded. A solvent correction was made for the presence of DMSO in the running buffer for experiments with inhibitors. Data were calculated using the Biacore T200 evaluation software 2.0 (GE Healthcare).

### Isothermal titration calorimetry (ITC) assay

Microcalorimetric measurement of the binding of IMB-YH-8 to PknB was performed on a MicroCal™ iTC 200 (GE Healthcare, USA) with 30 μM protein solution in the cell and 1200 μM of the compound solution in the syringe. Experiments were carried out at 25 °C in 25 mM Tris-Cl buffer containing 75 mM NaCl and 10% glycerol at pH 7.4 with a final DMSO concentration of 0.7%, in the syringe and the cell. The titration experiments were performed at 19 injections with 2.5 min intervals, using 0.5 μL for the first injection and 2 μL for the remaining injections. All data were corrected for heat of dilution by subtracting the heat for the ligand titration into the buffer alone. Data analysis was performed with the Origin 7.0 software (MicroCal) using a single binding site model. The binding free energy change Δ*G* and the entropy change Δ*S* were calculated from the equation Δ*G* = −*RT*ln*K*_*a*_ = Δ*H* − TΔ*S*.

### Docking studies

The mitoxantrone bound crystal structure (PDB-ID: 2FUM) was selected for docking studies^24^. The binding mode for IMB-YH-8 towards the binding site of PknB was generated through molecular docking using Molecular Operating Environment (MOE) version 2009.10^35^. In general, the docking was performed through “DOCK” module in MOE using the alpha triangle placement method. Refinement of the docked poses was carried out using the force field refinement scheme and scored using both the affinity dG and London dG scoring system. The pose with the highest docking score was returned for further analysis.

### Electron microscopy

To determine whether IMB-YH-8 affects the morphology of *M*. *tuberculosis* H37Rv, scanning electron microscopy (SEM) was performed. H37Rv was grown to early log phase (optical density at 600 nm, ~0.2). Then 1 μg/ml IMB-YH-8 was added to treat cells, followed by incubation at 37 °C for 5 days. Cells were harvested by low-speed centrifugation and washed in 0.1 M phosphate buffer (pH 7.4). The cells were then fixed in 0.1 M phosphate buffer (pH 7.4) containing 2.5% glutaraldehyde and 0.5% paraformaldehyde. The samples were submitted to the Instrument Center of Institute of Microbiology, Chinese Academy of Sciences for further analysis. Observations were carried out using a SEM (SU8010; Hitachi, Tokyo, Japan). Untreated H37Rv were also processed as control.

### RNA isolation

The drug-treated (2.5 μg/ml IMB-YH-8), together with control (DMSO) mycobacterial cultures, were incubated at 37 °C for 4 h. Total RNAs were isolated by RiboPure^TM^-Bacteria Kit (Life Technology, AM1925) according to the manufacturer’s instructions. The quantity and quality of each RNA sample was determined using NanoDrop ND-2000 spectrophotometer (Thermo Scientific, Wilmington, DE) and Agilent 2100 Bioanalyzer (Agilent Technologies, Santa Clara, CA).

### Microarray Labeling, Hybridization and data analysis

The qualified RNA samples were labeled and hybridized on Agilent 4 × 44 K Custom design Gene Expression Microarray according to manufacturer protocol (Agilent, Santa Clara, CA). In brief, 100 ng of each RNA sample was amplified to cRNA and labeled with Cy-3 using Agilent Low Input Quick Amp Labeling Kit. The labeled samples were hybridized for 16 hours at 10 rotations per minute. After hybridization, the arrays were washed and scanned by Agilent scanner and then the image was analyzed by Agilent Feature Extraction v11.5. Data obtained from Feature Extraction were imported to GeneSpring GX version 13.1 (Agilent Technologies, Santa Clara, CA) for 75th percentile shift and baseline to median of all samples normalization.

### RT-PCR

Reverse transcription was performed with PrimeScript™RT reagent Kit with gDNA Eraser (Takara). Briefly, remaining traces of DNA were digested by treating 1 μg RNA with 1 μl gDNA Eraser for 2 min at 42 °C. Each 20-μl RT reaction contained 300 ng purified total RNA, 1 μl RT primers mix and 1 μl PrimeScript RT Enzyme Mix I, in a 1× buffer supplied by the manufacturer. The RT reactions were proceeded 15 min at 42 °C, 5 sec at 85 °C and then chilled on ice.

The qRT-PCR was performed in duplicate with a StepOnePlus Real-Time PCR System (Applied Biosystems) using SYBR Premix ExTaq II (Takara, Japan) with gene-specific primers (Table S1). The quantitative PCR conditions were 95 °C for 30 s; then 40 cycles of 95 °C for 5 s, 60 °C for 30 s. Results were normalized to the amount of *sigA* mRNA, as previously described^36^.

### Minimal Inhibitory Concentration (MIC) assay

The *M*. *tuberculosis* strains used in these studies included the laboratory strain H37Rv (ATCC 27294; American Type Culture Collection, Rockville, MD) and drug-resistant clinical isolates. All clinical isolates were obtained from the State Laboratory of Tuberculosis Reference of China. *M*. *tuberculosis* H37Rv or a clinical isolate was grown at 37 °C in Middlebrook 7H9 Broth (Difco Laboratories, USA) supplemented with 0.2% (vol/vol) glycerol, 0.05% (vol/vol) Tween 80, and 10% (vol/vol) oleic acid albumin dextrose catalase (OADC) (BD and Company, USA). The activities of compound against H37Rv and clinical isolates were determined using the microplate alamar blue assay (MABA), as described previously^37, 38^. The MIC was defined as the lowest concentration eliciting a reduction in fluorescence of ≥90% relative to the mean of replicate bacterium-only controls.

### Cytotoxicity assay

Human monocytic leukemia THP-1 cells were cultured in RPMI 1640 medium supplemented with 10% fetal bovine serum at 37 °C under 5% CO_2_. Cells were seeded into 96-well plates (4 × 10^4^ cells/well in 100 μl culture medium) in duplicate, and differentiated with 10 ng/ml Phorbol-12-myristate-13-acetate (PMA) for 24 h. The contents of the wells were replaced with fresh RPMI 1640 medium containing 10% FBS. Three-fold serial dilutions of the stock solutions resulted in final concentrations of 64 to 0.26 μg/ml with a volume of 100 μl. After incubation at 37 °C for 48 h, 10 μl of 5 mg/ml methyl-thiazolyldiphenyl-tetrazolium bromide (MTT) were added to each well. Then the plates were incubated for 4 h, after which the absorbance was read at 570 nm.

### Intracellular activity assay

THP-1 cells (4 × 10^5^ cells/well in 1 ml culture medium) were differentiated with 10 ng/ml PMA, and grown overnight in RPMI 1640 medium containing 10% FBS in 24-well plates. *M*. *tuberculosis* H37Rv cultures were passed through an 8 μm-pore-size filter to remove clumps, and diluted to infect macrophages at a multiplicity of infection of 10 bacteria per cell. Infection was carried out for 4 hours, followed by three times washing with fresh media to remove the extracellular mycobacteria. The medium was replaced daily with different concentrations of compounds (2, 1, and 0.5 μg/ml). After 3 days of incubation, the medium was removed and the macrophages were lysed with 200 μl of 0.1% sodium dodecyl sulfate. Then the lysates were diluted with fresh media and plated onto 7H11 plates supplemented with 10% OADC to measure colony forming units (CFU). The results from the trend analysis were analyzed using Analysis of Variance (ANOVA) followed by the Fisher’s Least Significant Difference (LSD) post hoc test, and analysis over time was performed using repeated-measures ANOVA followed by the Fisher’s LSD post hoc test. Differences were considered statistically significant at p < 0.05. SPSS 23.0 (StatSoft Inc., Tulsa, OK) was used for the statistical analysis.

## Electronic supplementary material

Supplementary information

## References

[1] World Health Organization. Global Tuberculosis Report 2016. http://www.who.int/tb/publications/global_report/en/ (2016).

[2] Zumla A, Raviglione M, Hafner R, von Reyn CF (2013). Tuberculosis. The New England journal of medicine.

[3] Zumla AI (2014). New antituberculosis drugs, regimens, and adjunct therapies: needs, advances, and future prospects. The Lancet. Infectious diseases.

[4] Koul A, Arnoult E, Lounis N, Guillemont J, Andries K (2011). The challenge of new drug discovery for tuberculosis. Nature.

[5] Wehenkel A (2008). Mycobacterial Ser/Thr protein kinases and phosphatases: physiological roles and therapeutic potential. Biochimica et biophysica acta.

[6] Kang C-M (2005). The Mycobacterium tuberculosis serine/threonine kinases PknA and PknB: substrate identification and regulation of cell shape. Genes & development.

[7] Av-Gay Y, Everett M (2000). The eukaryotic-like Ser/Thr protein kinases of Mycobacterium tuberculosis. Trends in microbiology.

[8] Shah IM, Laaberki MH, Popham DL, Dworkin J (2008). A eukaryotic-like Ser/Thr kinase signals bacteria to exit dormancy in response to peptidoglycan fragments. Cell.

[9] Fernandez P (2006). The Ser/Thr protein kinase PknB is essential for sustaining mycobacterial growth. Journal of bacteriology.

[10] Chawla Y (2014). Protein kinase B (PknB) of Mycobacterium tuberculosis is essential for growth of the pathogen *in vitro* as well as for survival within the host. The Journal of biological chemistry.

[11] Ortega C (2014). Mycobacterium tuberculosis Ser/Thr protein kinase B mediates an oxygen-dependent replication switch. PLoS biology.

[12] Young TA, Delagoutte B, Endrizzi JA, Falick AM, Alber T (2003). Structure of Mycobacterium tuberculosis PknB supports a universal activation mechanism for Ser/Thr protein kinases. Nature Structural & Molecular Biology.

[13] Xing Yun HB, Jian X (2014). The establishment and application of a high throughput screening assay for inhibitors of Mycobacterium tuberculosis protein kinase B. Microbiology China.

[14] Av-Gay Y, Jamil S, Drews SJ (1999). Expression and characterization of the Mycobacterium tuberculosis serine/threonine protein kinase PknB. Infection and immunity.

[15] Mieczkowski C, Iavarone AT, Alber T (2008). Auto-activation mechanism of the Mycobacterium tuberculosis PknB receptor Ser/Thr kinase. The EMBO journal.

[16] Duran R (2005). Conserved autophosphorylation pattern in activation loops and juxtamembrane regions of Mycobacterium tuberculosis Ser/Thr protein kinases. Biochemical and biophysical research communications.

[17] Villarino A (2005). Proteomic identification of M. tuberculosis protein kinase substrates: PknB recruits GarA, a FHA domain-containing protein, through activation loop-mediated interactions. J Mol Biol.

[18] Kinoshita E, Kinoshita-Kikuta E, Koike T (2009). Separation and detection of large phosphoproteins using Phos-tag SDS-PAGE. Nature protocols.

[19] Prisic S (2010). Extensive phosphorylation with overlapping specificity by Mycobacterium tuberculosis serine/threonine protein kinases. Proceedings of the National Academy of Sciences of the United States of America.

[20] Narayan A (2007). Serine threonine protein kinases of mycobacterial genus: phylogeny to function. Physiological genomics.

[21] Kuijl C (2007). Intracellular bacterial growth is controlled by a kinase network around PKB/AKT1. Nature.

[22] Hers I, Vincent EE, Tavare JM (2011). Akt signalling in health and disease. Cellular signalling.

[23] Luo Y (2005). Potent and selective inhibitors of Akt kinases slow the progress of tumors *in vivo*. Molecular cancer therapeutics.

[24] Wehenkel A (2006). The structure of PknB in complex with mitoxantrone, an ATP-competitive inhibitor, suggests a mode of protein kinase regulation in mycobacteria. FEBS letters.

[25] Ortiz-Lombardia M, Pompeo F, Boitel B, Alzari PM (2003). Crystal structure of the catalytic domain of the PknB serine/threonine kinase from Mycobacterium tuberculosis. The Journal of biological chemistry.

[26] Takayama K, Wang L, Merkal RS (1973). Scanning electron microscopy of the H37Ra strain of Mycobacterium tuberculosis exposed to isoniazid. Antimicrobial agents and chemotherapy.

[27] Tandon R (2011). Characterization of 7-amino-4-methylcoumarin as an effective antitubercular agent: structure-activity relationships. The Journal of antimicrobial chemotherapy.

[28] Khan S (2010). Phosphorylation of enoyl-acyl carrier protein reductase InhA impacts mycobacterial growth and survival. The Journal of biological chemistry.

[29] Veyron-Churlet R, Zanella-Cleon I, Cohen-Gonsaud M, Molle V, Kremer L (2010). Phosphorylation of the Mycobacterium tuberculosis beta-ketoacyl-acyl carrier protein reductase MabA regulates mycolic acid biosynthesis. The Journal of biological chemistry.

[30] Park ST, Kang CM, Husson RN (2008). Regulation of the SigH stress response regulon by an essential protein kinase in Mycobacterium tuberculosis. Proceedings of the National Academy of Sciences of the United States of America.

[31] Raman S (2001). The alternative sigma factor SigH regulates major components of oxidative and heat stress responses in Mycobacterium tuberculosis. Journal of bacteriology.

[32] Lougheed KE (2011). Effective inhibitors of the essential kinase PknB and their potential as anti-mycobacterial agents. Tuberculosis.

[33] Sassetti CM, Boyd DH, Rubin EJ (2003). Genes required for mycobacterial growth defined by high density mutagenesis. Molecular microbiology.

[34] Zhai Q (2015). Validated LC-MS/MS method for determination of YH-8, a novel PKnB inhibitor, in rat plasma and its application to pharmacokinetic study. Acta pharmaceutica Sinica. B.

[35] Molecular Operating Environment (MOE). Montreal, Canada:Chemical Computing Group Inc., 2009.10.

[36] Manganelli R, Dubnau E, Tyagi S, Kramer FR, Smith I (1999). Differential expression of 10 sigma factor genes in Mycobacterium tuberculosis. Molecular microbiology.

[37] Collins L, Franzblau SG (1997). Microplate alamar blue assay versus BACTEC 460 system for high-throughput screening of compounds against Mycobacterium tuberculosis and Mycobacterium avium. Antimicrobial agents and chemotherapy.

[38] Lu Y (2011). Clofazimine analogs with efficacy against experimental tuberculosis and reduced potential for accumulation. Antimicrobial agents and chemotherapy.

