# Supplementary material for: A novel protein kinase inhibitor IMB-YH-8 with anti-tuberculosis activity
Source: Sci Rep. 2017 Jul 11;7:5093. doi: 10.1038/s41598-017-04108-7 (PMC5506005; doi:10.1038/s41598-017-04108-7)
Supplement: Supplementary file 1 — Supplementary information [file 41598_2017_4108_MOESM1_ESM.pdf]

A novel protein kinase inhibitor IMB-YH-8 with anti-tuberculosis activity

Jian Xu[1,2],Ju-xian Wang[1], Jin-ming Zhou[1], Chang-liang Xu[1], Bin Huang[1], Yun Xing[1],Bin Wang[2], Rui Luo[1], Yu-cheng Wang[1], Xue-fu You[1], Yu Lu[2#]and Li-yan Yu[1#]

[1]Institute of Medicinal Biotechnology, Chinese Academy of Medical Sciences and Peking Union Medical College, 1# Tian Tan Xi Li, Chongwen district, Beijing 100050, China

[2]Beijing Key Laboratory of Drug Resistance Tuberculosis Research, Beijing Tuberculosis and Thoracic Tumor Research Institute, Beijing Chest Hospital, Capital Medical University, Beijing 101149, China

#Corresponding authors.

Li-yan Yu, Tel: +86-010-63187118; Fax: +86-010-63187118; E-mail: yly@cpcc.ac.cn .

Yu Lu, Tel: +86-010-89509357; Fax: +86-010-80505770; E-mail: luyu4876@hotmail.com .

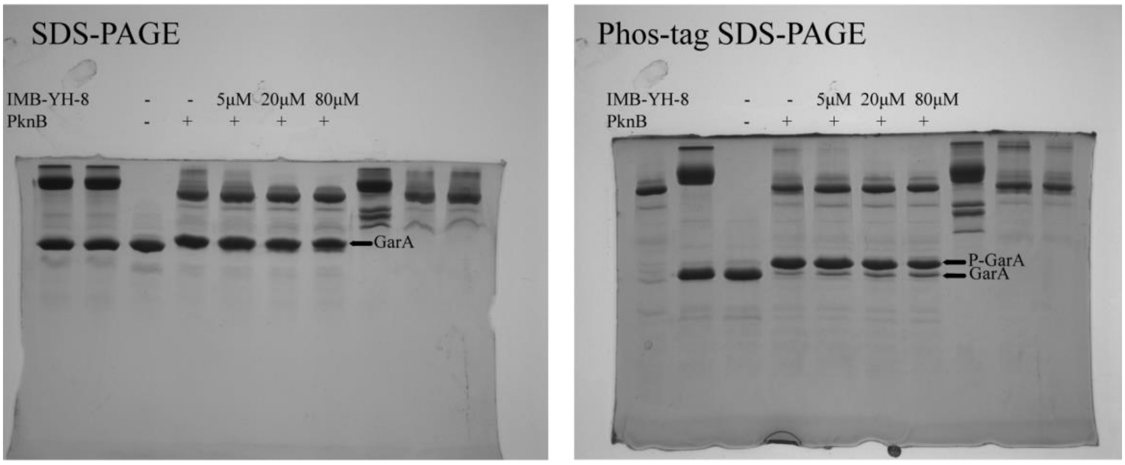

Supplementary Figure 1: *In vitro* activity of IMB-YH-8 in substrate phosphorylation of GarA by PknB (full-length gels)

Table S1: Gene specific primers used in this study

**Gene primers**	**Sequence**
Rv3914	F: 5'-GAAGTCCGCCACCATCAA-3'
	R: 5'-GTCGCCCAAAAGTCAACC-3'
Rv3913	F: 5'-TGCTGGTTGACTTTTGGG-3'
	R: 5'-GCGACGGTGAGGTCTGTT-3'
Rv2466c	F: 5'-AGTCTGTCGCCGATTTCT-3'
	R: 5'-CAGGTCGTCACGGTTTTC-3'
Rv3221A	F: 5'-GACCACGACGATTCCCACG-3'
	R: 5'-AGACGCTCCCGCAAGCCCTC-3'
Rv3223c	F: 5'-ACAGACGAGGAGTTGACC-3'
	R: 5'-GAACGAAATCCCGCATAG-3'
SigA	F: 5'-CGCGAAAAACCATCTG-3'
	R: 5'-GATCAGCCCCAGGTTG-3'
